# The Use of Sage Oil Macerates (*Salvia officinalis* L.) for Oxidative Stabilization of Cod Liver Oil in Bulk Oil Systems

**DOI:** 10.1155/2020/4971203

**Published:** 2020-12-16

**Authors:** Agnieszka M. Hrebień-Filisińska, Artur Bartkowiak

**Affiliations:** ^1^Department of Fish, Plant and Gastronomy Technology, Faculty of Food Sciences and Fisheries, West Pomeranian University of Technology, 71-459 Szczecin, Poland; ^2^Center of Bioimmobilisation and Innovative Packaging Materials, Faculty of Food Sciences and Fisheries, West Pomeranian University of Technology, 71-270 Szczecin, Poland

## Abstract

The aim of the study was to investigate the antioxidant properties of sage oil macerates (M) in cod liver oil (CLO) during process oxidation catalyzed by UV radiation. CLO was not only subject to oxidative stabilization but also used as a solvent for active ingredients of sage. Macerates were obtained by combining the sage with CLO, homogenization, maceration, and filtration. The effect of different maceration times (0, 3, 6, 8, 10, 13, and 15 days) and different concentrations of macerate addition (5%, 10%, 25%, and 50%) on the CLO oxidation degree, which was determined by peroxide value (PV), anisidine value (AV), and Totox index, was evaluated. Additionally, the total content of polyphenols in macerates by the Folin-Ciocalteu method, antioxidant activity DPPH, and color was determined. The macerates showed antioxidant properties in CLO. The best effect was shown by the initial macerate (maceration time 0, M_0_), which in 25% concentration significantly inhibited oxidative processes in CLO. It was also characterized by high content of polyphenols and antioxidant activity of DPPH. Sage macerates can effectively inhibit oxidation of fish oils and prolong their durability.

## 1. Introduction

Due to the presence of unsaturated omega-3 fatty acids, especially long-chain EPA (eicosapentaenoic acid) and DHA (docosahexaenoic acid), fish oils are beneficial for human health. At the same time, they are very unstable and easily subject to oxidative processes, which reduce their nutritional value. The use of synthetic antioxidants is increasingly often questioned [1]. Therefore, tocopherols are commonly used to stabilize fish oils [2–4]. However, tocopherols do not always show high antioxidant effects [2], particularly at elevated temperatures, where they may degrade [5]. Therefore, they are often used in combination with other antioxidants, usually in the form of a three-component composition with ascorbyl palmitate and lecithin [6] or with ascorbyl palmitate and citric acid [7]. The combinations of tocopherols, lecithin, and ascorbyl palmitate with rosemary extract [6] and L-lysine [8] are also applied. Moen et al. [9] elaborated a mixture of antioxidants based on tocopherols, ascorbyl palmitate, rosemary extract, and green tea catechins for the preservation of omega-3 marine oil concentrates.

New sources of the so-called natural antioxidants are also sought after among plant polyphenols. The antioxidant role of polyphenols (AH) consists mainly in capturing free radicals of LOO∗ with the production of a nonradical product (A∗) [10]:

(1) $$\begin{array}{c} \text{LOO}*+\text{AH}⟶\text{LOOH}+\text{A}*. \end{array}$$

Polyphenols have an antioxidant effect on the initial oxidation process and against radicals formed in later stages of the oxidation process [11].

Polyphenolic compounds obtained from by-products of the fruit and vegetable industry [12–17], cactus pear [18], barley [19, 20], blueberry [21], strawberry leaves [22], legumes [23], rice wine [24], ground coffee [25], and seaweed (*Kappaphycus alvarezii*, *Hypnea musciformis*, and *Jania rubens*) [26] were used in the studies for oxidative stabilization of fish oils. Some isolated compounds of plant origin such as caffeic acid [27–29], quercetin, catechins, morin and myricetin [30–34], hydroxytyrosol [35], sesamol [36], and curcumin [37] were also used.

The rich sources of polyphenols are spices and herbs, which in addition to antioxidant effect, also exhibit antibacterial, antifungal, and anti-inflammatory properties. Rosemary [29, 38–41] and rosemary in combination with other antioxidants [6, 42–44] were usually used for inhibition of oxidation processes in fish oils. Rarely, oregano [39, 41], cloves [45], or sage extracts [38] were added to fish oils. Among herbs, rosemary is the best known antioxidant additive. It is also the only plant species which is considered in the EU as authorized food additives with the possibility of their use in fish oils (Commission Regulation (EU) No. 1129/2011 of 11 November 2011).

Attention should be paid also to the medicinal sage (*Salvia officinalis* L.), which although known for its antioxidant and bactericidal properties and despite its similarity to rosemary, is less commonly used to stabilize fish oils. Recently, also a significant anticancer activity has been attributed to sage [46].

The antioxidant activity of sage is mainly related to the content of active compounds in the plant. It also depends on the extraction process conditions and the type of solvent used [47], as it is most often used in the form of extracts.

Organic solvents, supercritical carbon dioxide, and edible oils are most commonly used for the extraction of active ingredients [48]. The application of edible oils is very simple and safe. This method has many advantages, e.g., it does not pose problems related to evaporation after solvent extraction and its residue [48]. Carnosolic acid, one of the main components responsible for sage antioxidant activity, is characterized by an increased lipid solubility [49] and shows high persistence and stability in fats [50–52], as opposed to polar solvents, in which it may be perishable and may undergo degradation processes. Therefore, the use of oils for the extraction of antioxidant components from sage may prove to be effective and the addition of such extracts to fish oils may inhibit their oxidation and prolong their durability.

In this study, an attempt was made to use cod liver oil to extract polyphenols from sage. Cod liver oil was used as a solvent for active ingredients in sage and was also subject to oxidative stabilization. The aim of the study was to evaluate the effect of sage oil macerates on oxidative changes of fish oil during oxidation catalyzed by UV radiation.

## 2. Materials and Methods

### 2.1. Cod Liver Oil

The study was carried out on cod liver oil (LYSI, Iceland). The oil without antioxidant additives was purchased and delivered in 22 kg barrels. It was stored in a cold store at temperature 4-5°C and used before the expiry date. After taking a portion of oil from the barrel for analysis, the barrel was filled with nitrogen and closed tightly.

### 2.2. Sage (*Salvia officinalis* L.)

Sage (*Salvia officinalis* L.) in dry and crushed form was obtained from a Polish herb producer. According to the producer's declaration, the samples of sage were taken from the harvest of Bona variety. Before the study, the dry plant material was ground in a mill to a powdered form, with a particle diameter of up to 0.4 mm, and then stored in tight plastic bags in a dark place until analysis, but not longer than four weeks.

### 2.3. Sage Macerate Obtaining

**Figure 1:**
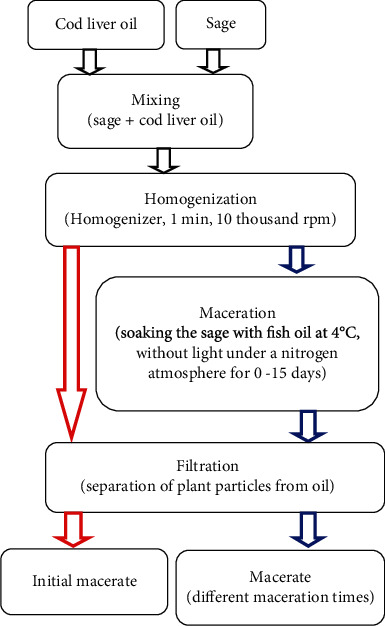
Formation of macerates.

The oil macerate of sage was obtained by mixture of sage with cod liver oil (CLO), homogenization, maceration (using different maceration times), and then separation of the oil phase from plant particles (see Figure 1).

Ground sage was homogenized with CLO for 1 minute (rotational speed 10000 rpm) using a knife homogenizer (POL-EKO, Poland). The sage to fish oil typical ratio was 1/5.7; other weight ratios were also examined: 1/9 and 1/12. The suitable weight ratios of sage to fish oil were experimentally determined in preliminary studies. The ratio of 1/5.7 was the highest possible ratio of sage to oil with optimal wetting of dried sage by oil. Additionally, two lower weight ratios 1/9 and 1/12 have been tested.

The obtained system was placed in a glass bottle, covered with aluminum foil, filled with nitrogen, sealed, and stored at 4°C for maceration. The samples were collected for analysis on the day of macerate preparation (in case of M_0_ initial macerate) and after different days of maceration. For this purpose, the bottle with sage and oil was shaken vigorously, and portions of the raw macerate M_C_ were taken and seeped through a Büchner funnel (medium softness filter) using a vacuum pump. The bottle was refilled with nitrogen, sealed, and stored until the end of the experiment. In turn, the already purified macerate (M), after the separation of plant particles, was used to stabilize the fish oil, and analyses were carried out.

### 2.4. Research and Methods Applied

The antioxidant properties of the obtained macerates were examined. Each macerate (initial M_0_ and M after different maceration times—after 3, 6, 8, 10, 13, and 15 days of storage) was added to cod liver oil in four different weight concentrations (*w*/*w*): 5%, 10%, 25%, and 50%, and the antioxidant effect has been tested during accelerated oxidation with UV light. The effects of maceration time and sage to the CLO weight ratio on polyphenol extraction, DPPH antioxidant activity, color, and oxidation degree were also studied. Additionally, sensory analysis of cod liver oil with 25% addition of macerate M_0_ was performed.

### 2.5. Accelerated Oxidation Test

The samples of cod liver oil (10 g) with different macerate concentrations were homogenized for 1 minute (10000 rpm, homogenizer—POL-EKO, Poland), poured into crystallizers (diameter × height: 50 × 30 mm), and exposed to UV rays for 60 minutes (UV lamp: Honle, Germany, UVASPOT 400T, emitting UVA+VIS in the range from 315 nm to 400 nm, filter H1). The distance between the samples and the lamp was 14 cm. During the irradiation, there was a gradual increase in temperature of the samples placed under the lamp, which after 30 minutes reached about 72°C and after 60 minutes about 87°C. Then, the oil samples were dissolved in chloroform, and the oxidation level was determined using the titration peroxide value (PV), anisidine value (AV), and Totox index. Analyses were also carried out for exposed cod liver oil (CLO_UV_) and for unmodified oil, not exposed to irradiation—the initial oil (CLO_0_).

### 2.6. Determination of Oxidation Level

Titration peroxide value (PV) in meqO/kg fat was determined according to standard [53]. 15 cm^3^ of acetic acid and 1 cm^3^ of saturated potassium iodide were added into flasks with oil samples dissolved in chloroform; then, the flasks were closed, stirred for 1 min, and placed for 5 min in a dark place. Then, 75 cm^3^ of water was added, and the secreted iodine 0.002 N sodium thiosulfate was titrated in the presence of several drops of starch solution.

Anisidine value (AV) was determined according to standard [54]. 1 cm^3^ of anisidine reagent (prepared from p-anisidine and glacial acetic acid according to the standard procedure) was added to oil samples dissolved in chloroform, and after mixing, the sample was left in a dark place. After 10 minutes, the absorbance was measured at *λ* = 350 nm with respect to the blank test.

The Totox index was calculated according to standard [54] from the formula

(2) $$\begin{array}{c} \text{Totox}=2\times \text{LN}+\text{LA}. \end{array}$$

### 2.7. Delta Factor (*Δ*)

To assess the antioxidant efficacy, the delta factor (*Δ*) was used, which was calculated for the peroxide value, anisidine value, and Totox index using the following formula:

(3) $$\begin{array}{c} \Delta =\frac{\left(A-A0\right)\times 100\%}{A1-A0}, \end{array}$$

where *A* is the result of the examined sample (fish oil with macerate), *A*0 is the result for the initial fish oil without irradiation, and *A*1 is the result for the fish oil exposed to UV.

The formula is an analogous reflection of the CL indicator used by Burkow et al. [38] in the chemiluminescent method to assess the antioxidant efficiency of plant extracts in cod liver oil. This factor shows the percentage share of changes in the fish oil under the influence of UV in relation to the initial fresh fish oil (unexposed).

### 2.8. Extraction of Active Compounds (Polyphenols) from Macerate to the Hydrophilic Phase

In order to determine the polyphenols and DPPH activity in sage oil macerate, these compounds were first extracted from the hydrophobic phase (macerate) to the hydrophilic phase (methanol solution), for which appropriate analyses were carried out.

The extraction was made according to Zadernowski et al. [55], replacing mechanical shaking with ultrasound extraction. Macerate (5 g) was dissolved in hexane (20 cm^3^) and shaken for 1 min with 70% methanol (100 cm^3^); then, the samples were extracted for 5 minutes in an ultrasonic bath (Ultrasonic bath SB-3200DTD, China), maintaining the following parameters: frequency -40 kHz, power -180 W, and process temperature 20°C. Then, the samples were shaken in a glass separator, and after phase separation, the methanol layer was centrifuged (15 min) in a centrifuge (MPW 223-e, Poland; 4000 rpm). Polyphenol content and antioxidant activity DPPH were determined in the obtained methanol extract.

### 2.9. Determination of Polyphenols and Antioxidant Activity DPPH in Macerates

The total content of polyphenols was determined according to Singleton and Rossi [56] by the spectrophotometric method with the Folin-Ciocalteu reagent in the presence of caffeic acid as a standard. The Folin-Ciocalteu reagent (5 cm^3^) and saturated calcium carbonate (10 cm^3^) were added to 5 cm^3^ of the obtained methanol-water extract; after water addition to 100 cm^3^ and mixing of the whole, the samples were left in a dark place. After 1 hour, the absorbance of the blue solution was measured at *λ* = 760 nm. A standard curve was also made for different concentrations of caffeic acid solution. The results are presented in mg of polyphenolic compounds converted into caffeic acid in 100 g of macerate.

The antioxidant activity DPPH was determined according to Yen and Chen [57]. The principle consists in colorimetric measurement of the degree of DPPH (1,1-diphenyl-2-picrylohydrazil) free radical reduction in the extracts. DPPH solution was prepared by dissolving DPPH radicals in methanol. 3 cm^3^ of methanol and 1 cm^3^ of DPPH solution were added to 1 cm^3^ of diluted extract (1 : 9), and after mixing, the sample was set aside in a dark place. After 10 minutes, the absorbance of samples and the blank sample was measured with respect to methanol at *λ* = 517 nm. The percentage of DPPH inhibition was calculated on the basis of the formula by Rossi et al. [58]:

(4) $$\begin{array}{c} \%\text{DPPH}=100-\left(\frac{A_{p}}{A_{0}}\right)\times 100, \end{array}$$

where *A*_*p*_ is the absorbance of the examined sample and *A*_0_ is the absorbance of the blank sample 650 nm.

### 2.10. Color Determination

The color was determined according to standard [59] using the formula

(5) $$\begin{array}{c} \text{Color}=1000\times \left(A_{442}+A_{668}\right), \end{array}$$

where *A*_442_ is the absorbance of 1 cm^3^ macerate and 10 cm^3^ hexane at *λ* = 442 nm (carotenoids) and *A*_668_ is the absorbance of 3 cm^3^ macerate and 3 cm^3^ hexane at *λ* = 668 nm (chlorophyll).

Measurements of absorbance in the macerate samples (in each case) were determined with respect to the blank sample prepared on the basis of cod liver oil.

### 2.11. Determination of Polyphenols, Antioxidant Activity DPPH, and Carotenoids in Sage

The polyphenols were extracted with 70% methanol (40 cm^3^) from the dry and ground sage in a water bath under a condenser for half an hour. After cooling, the samples were filtered and supplemented with 70% methanol to 100 cm^3^, and thus, an extract was obtained. The total content of polyphenols was determined according to Singleton and Rossi [56] by a spectrophotometric method with the Folin-Ciocalteu reagent in the presence of caffeic acid as a standard, as in the case of macerates. The results have been presented in mg of polyphenolic compounds in 100 g d.m. sage expressed as caffeic acid.

The antioxidant activity of DPPH in sage was determined according to Yen and Chen [57], analogously to the determination in macerate. Sage extracts were obtained by extracting the sage with methanol for 5 minutes in an ultrasonic bath. The extract was diluted 1 : 9 for determinations.

Total carotenoids were determined using the methodology of Lichtenthaler and Wellburn [60]. The method is based on the extraction of sage with 80% acetone in an ultrasonic bath for 5 minutes (ultrasonic bath SB-3200DTD, China, frequency -40 kHz, power -180 W, and process temperature 20°C). The absorbance of the centrifuged extract was then measured at three wavelengths, 441, 646, and 663 nm, respectively. The result was quantified in mg of total carotenoids in kg d.m. of sage.

### 2.12. Sensory Analysis

Sensory analysis of cod liver oil with 25% macerate was performed by a team of 5 persons moderately experienced in sensory evaluation. The participants were tested for sensory sensitivity in standards [61, 62]. Before starting the analysis, the team was well instructed on the principles of the study. Two initial training sessions were conducted prior to the relevant tests. In order to examine the influence of the indicated additives on the change of taste and smell of CLO, a profile method was used, assessing the taste and smell of fish oil according to the method described in the ISO standard procedure. The intensity of the individual characteristics in the profile method was evaluated on a 10-point scale (where 0 is undetectable and 10 is very noticeable).

### 2.13. Statistical Analysis

The nonparametric Kruskal-Wallis test was used to assess the significance of differences between the groups, which is the equivalent of one-factor analysis of variance (ANOVA) (the analyzed variables did not meet the conditions required to perform parametric tests—lack of uniformity of variance). Spearman's rank correlation coefficient was used to assess the correlation between the variables. The significance of the analyzed values was assumed for *p* < 0.05. The results were statistically elaborated using the STATISTICA 9.0 program.

## 3. Results and Discussion

### 3.1. Characteristics of Medicinal Sage

Sage used in the production of macerates contained antioxidant components: polyphenols and carotenoids and showed antioxidant activity DPPH against free radicals (see Table 1).

The antioxidant capacity of polyphenols results from the presence of hydroxyl groups connected with an aromatic ring [63]. The mechanism of their action consists in the capturing of free radicals of LOO∗ and production of a nonradical product [10]. Carotenoids, on the other hand, can act as effective 1O_2_ singlet oxygen extinguishers, which are associated with the presence of at least 9 conjugated double bonds [64].

### 3.2. Characteristics of Macerates

The effect of maceration time on polyphenol content in macerate was found, but no effect of maceration time on DPPH antioxidant activity was observed (see Table 2). In the case of polyphenols, on the 15th day of maceration, there was a significant increase in their content compared to the sample immediately after homogenization. However, those polyphenols which passed to the oil phase during maceration did not show an increased free radical scavenging activity in comparison to those extracted after homogenization and filtration (initial macerate M_0_), as the DPPH activity both in the initial macerate and after each maceration time remained constant. It is likely that the process of substance extraction took place until its concentration in the sage tissue balanced the concentration of the substance dissolved in the solvent (in this case CLO), unless the extraction was additionally limited by solubility [65]. Probably, the polyphenolic compounds with lyophilic properties, which are soluble in oils, passed to oil most quickly during maceration. Therefore, large amounts of carnosic acid and its derivatives can be expected in the macerate, because carnosic acid has lipophilic properties [66] and is characterized by increased lipid solubility [49].

Sage is a special source of antioxidants because it contains polyphenols with a wide range of polarity and solubility, with carnosic acid being on the lipophilic end of the scale and rosmarinic acid on the hydrophilic end [66].

Jović et al. [67] performed maceration from herbs (including sage) based on olive oil with ultrasound homogenization. For comparison, the maceration process was conducted also with water. The obtained products have a high concentration of polyphenols, carotenoids, and chlorophyll. It was observed that the difference in polyphenol content between water and oil macerates was very low, which may be due to the hydrophilic properties of the active ingredients of sage. However, in both cases extracted polyphenols due to their potential various chemical structures may have different antioxidant properties.

The effect of maceration time on the content of pigments in the macerate was also found, which was manifested in a significant increase in color on the 9th and 15th days of maceration. The high value of the color parameter is the result of chlorophyll and carotenoid extraction. Sage, like other plants, contains photosynthetic dyes in its green parts, which passed to the oil phase during the maceration.

The mass ratio of sage to fish oil used in macerate production was 1/5.7, and it was the highest possible ratio of sage to fish oil at which the best total wetting of the dried sage was achieved. Other ratios were also tested: 1/9 and 1/12; however, in the case of 1/5.7, the highest total polyphenol content and the highest antioxidant activity DPPH in the oil phase were obtained (Table 3). A significant correlation between polyphenol content and DPPH activity was observed. Spearman's correlation coefficient was 0.899 (at *p* < 0.05). Similarly, other studies [68, 69] showed a high correlation between DPPH activity and total polyphenol content. It was also observed in the study that color substances were the most extracted from sage at the reagent ratio of 1/5.7 (see Table 3).

The process of maceration obtaining caused oxidative changes in the macerate (see Table 4). After preparation, the macerates were characterized by an increased content of primary oxidation products (PV increase) compared to the CLO used to prepare the macerate (see Table 4). The higher peroxide value resulted in a high Totox index. On the other hand, the preparation process did not affect the content of secondary oxidation products; hence, the AV increased significantly.

It is likely that the oxidation of the macerate could have been influenced by the preparation process mainly homogenization and the presence of chlorophyll. Due to the high content of unsaturated omega-3 fatty acids, cod liver oil is very labile and prone to oxidation processes [11]. Oxidation of oils occurs during the contact of fatty acids with atmospheric oxygen, where light, presence of metals, and increased temperature may accelerate these changes [70]. Homogenization, which was carried out at room temperature, may have facilitated the contact of the liver with oxygen and contributed to its oxidation. Moreover, due to the presence of chlorophyll in the macerate, despite short contact with daylight, photooxidation may have occurred during processing. In the presence of light and atmospheric oxygen, chlorophyll may act as a photosensitizer and may contribute to the initiation of oxidation processes, usually by excitation of singlet oxygen [31, 64, 71]. Another cause of oxidation in the initial matrix may have been impurities from sage. During harvest and processing, agricultural products are exposed to a wide range of microbiological contamination. The bacteria may be the source of lipoxygenase [72], an enzyme catalyzing the oxidation of polyunsaturated PUFA acids of the cis,cis-1,4-pentadiene arrangement with molecular oxygen [71].

### 3.3. Stabilization of Cod Liver Oil

**Figure 2:**
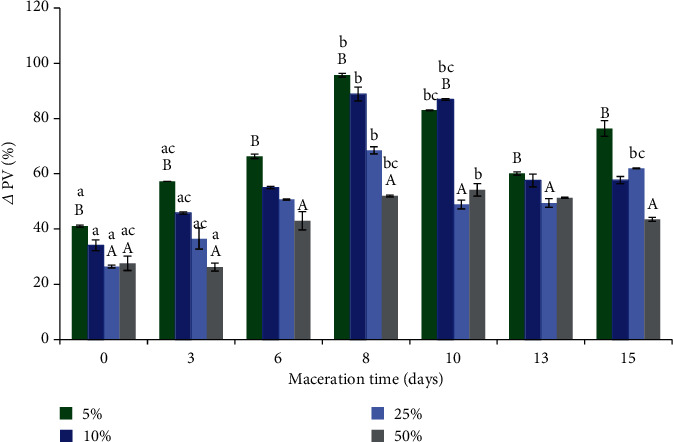
The effect of the addition of different concentrations of sage macerate (5%M-50%M), applied after a different day of maceration, on CLO quality parameters, expressed as delta (*Δ*) of peroxide value (PV), in the UV test (Values marked with the same lowercase letter a, ac,…, do not differ statistically significantly (*p* < 0.05) within each macerate concentration, while the values marked with the same uppercase letter—A, AC—do not differ statistically significantly (*p* < 0.05) on individual maceration days).

**Figure 3:**
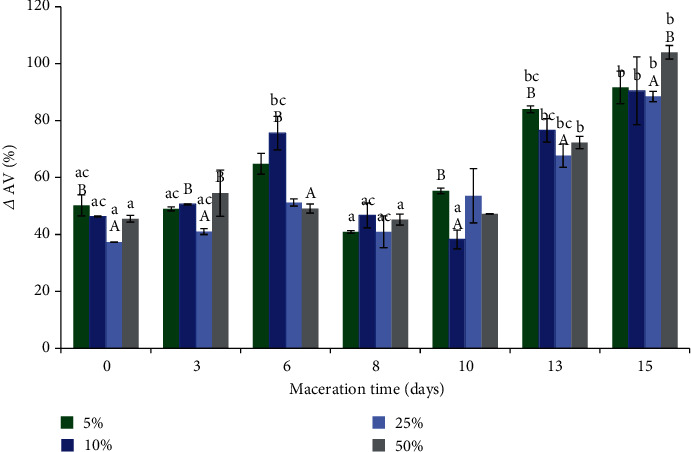
The effect of the addition of different concentrations of sage macerate (5%M-50%M), applied after a different day of maceration, on CLO quality parameters, expressed as delta (*Δ*) of anisidine value (AV), in the UV test (Values marked with the same lowercase letter a, ac,…, do not differ statistically significantly (*p* < 0.05) within each macerate concentration, while the values marked with the same uppercase letter—A, AC—do not differ statistically significantly (*p* < 0.05) on individual maceration days).

**Figure 4:**
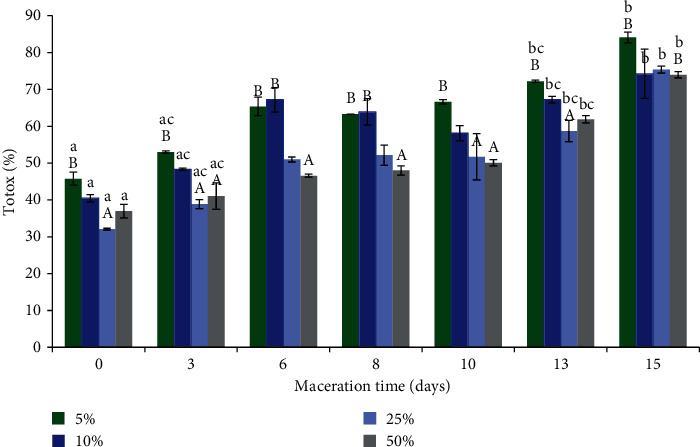
The effect of the addition of different concentrations of sage macerate (5%M-50%M), applied after a different day of maceration, on CLO quality parameters, expressed as delta (*Δ*) of Totox, in the UV test (Values marked with the same lowercase letter a, ac,…, do not differ statistically significantly (*p* < 0.05) within each macerate concentration, while the values marked with the same uppercase letter—A, AC—do not differ statistically significantly (*p* < 0.05) on individual maceration days).

Macerate, despite the presence of primary oxidation products, showed an antioxidant effect on the accelerated UV oxidation test when added to CLO (see Figures 2–4). It was found that the best antioxidant effect in cod liver oil was shown by the addition of 25% macerate (see Figures 2–4). As the maceration time was prolonged and an increasingly older macerate (almost in every case of the amount of macerate added to the fish oil, i.e., 5, 10, 25, and 50%) was added to CLO, the efficiency of inhibition of oxidation processes decreased and more primary and secondary oxidation products were accumulated (a clear increase in Totox index, see Figure 4). The prolongation of maceration time did not increase the amount of polyphenols active with respect to free radicals in the macerate but only increased the content of color components, including chlorophyll, which most probably explains the decrease of antioxidant effect with the prolonged maceration time during the UV test. Chlorophyll can act as a catalyst for oxidation in the presence of light [31, 64, 71]; the UV radiation used in the accelerated fish oil ageing test intensified the oxidative changes.

Analyzing different macerate contents in the oil, almost after each day of maceration, it was observed that the higher the macerate content in the oil, the lower the level of primary oxidation products (lower PV), while with an increase in macerate concentration in the fish oil to 25%, the content of secondary oxidation products decreased (lower AV). However, further increase of macerate concentration up to 50% resulted in a significant increase of secondary oxidation products (see Figures 2–4).

The antioxidant effect of sage preparations with respect to primary oxidation products in cod liver oil was obtained by Burkow et al. [38]. In the study where they tested various antioxidants, sage extract on an oil carrier inhibited the formation of hydronadoxides determined by the chemiluminescent (CL) method; however, the experiment with Rancimat did not confirm the effectiveness of sage extract against the secondary oxidation products.

The reduction of the peroxide value together with the increasing content of macerate in the oil could have been related to the higher concentration of polyphenols, which showed their antioxidant effect against the primary oxidation products. The high content of macerate in fish oil (50%), which caused an increase in secondary oxidation products (increase in AV), was probably related to the quality of the macerate itself, the present peroxides, and chlorophyll, which at this macerate concentration in fish oil caused negative changes. During the 50% addition of macerate to cod liver oil, peroxides introduced simultaneously in a larger amount during UV irradiation probably transformed into aldehydes and ketones, which caused an increase in the anisidine value. Furthermore, the peroxides present in the macerate may have catalyzed further oxidation reactions [64].

The maceration time did not significantly increase the content of polyphenols in the macerate (exception 15th day of maceration); however, at the same time, it enhances the content of plant pigments, including chlorophyll. Therefore, the best antioxidant effect on the UV acceleration test was obtained after an application of the initial macerate (M_0_), at 25% (*w*/*w*). Such amount of macerate contained an appropriate concentration of antioxidant polyphenols, which showed their effect and at the same time the smallest possible amount of prooxidants (chlorophylls).

Similar to the analyzed macerates of sage, Bracco et al. [73] used groundnut oil. Antioxidants dissolved in the oil lipid phase were then separated by two-stage molecular distillation and used to stabilize chicken fat. They inhibited oxygen absorption and showed better effects than rosemary, BHT, cocoa shell extract, and control. In this study, an attempt was made to use cod liver oil alone to extract polyphenols from sage. Cod liver oil was used as a solvent for active ingredients in sage and was also subject to oxidative stabilization. The use of plant oils for extraction, although they are more stable than fish oils [74], could change the profile of fatty acids due to dilution of valuable EPA and DHA during fish oil stabilization with such plant macerate.

### 3.4. Sensory Analysis

**Figure 5:**
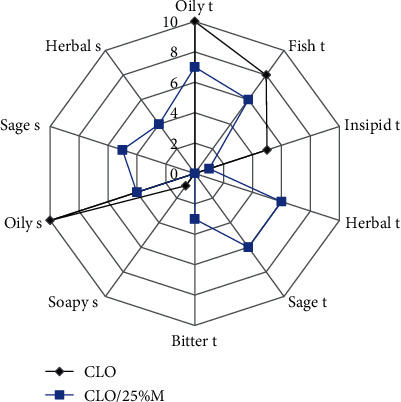
Taste (t) and smell (s) profile of fish oil with 25% sage macerate (CLO/25%M) in comparison to raw fish oil CLO.

The macerate added to the fish oil improved the taste, as it reduced the intensity of the fish and insipid taste and made the herbal and sage taste slightly noticeable (see Figure 5). The smell of fish oil after the addition of macerate was different from the typical smell of fish oil, and a slight herbal and sage flavor appeared.

Such methods are known in the literature to improve not only the stability but also the taste of plant oils. In the study, by the addition of various aromatic herbs, the palatability and stability of olive oil were enriched to varying degrees [75, 76]. Recently, flavorful plant oils with the addition of various spices have been frequently found as commercial products, which are popular among a large group of consumers.

## 4. Conclusions

Sage oil macerates were characterized by antioxidant properties. The best antioxidant effect in cod liver oil in the UV acceleration test was obtained after an application of the initial macerate (M_0_), at 25% (*w*/*w*). During macerate preparation, the active molecules passed from sage to oil already during the homogenization process without the need to store it after for more efficient extraction. The prolongation of maceration time was conducive to extraction of plant pigments. At the highest proportion of sage to fish oil (1/5.7), where good wettability of drought was maintained, high levels of active ingredients were obtained. The intensity of typical fish and liver taste and smell has been reduced in favor of herbal and sage flavor. Sage oil macerates can effectively inhibit the oxidation of fish oils and extend their shelf life. They are completely natural, safe, and characterized by a low-processing degree. In addition to the antioxidant effect, the possibility of combining EPA and DHA fatty acids with plant polyphenols may allow obtaining a natural composition of compounds with special health properties, not raising objections from the point of view of consumer safety.

## Figures and Tables

**Table 1:** Dry matter and selected plant components of sage.

Dry matter (%)	Carotenoids (mg/kg d.m.)	Polyphenols (mg/100 g d.m.)	DPPH activity (%)
92.1 ± 0.10	775 ± 37	7141 ± 83	10.3 ± 1.06

**Table 2:** Polyphenol content, antioxidant activity DPPH, and color in the macerate depending on the time of maceration.

Maceration time (days)	Polyphenols (mg/100 g)	DPPH (%)	Color
0 (day of preparation)	161.4 ± 5.46^b^	24.6 ± 1.40	835 ± 3.54^b^
2	159.5 ± 1.52^b^		1230 ± 1.41
4	160.6 ± 12.1	24.2 ± 1.22	1192 ± 28.3^bc^
7	162.7 ± 4.86	25.5 ± 1.07	1329 ± 21.9
9	170.2 ± 0.30		1378 ± 61.5^ac^
15	183.3 ± 5.46^a^	25.3 ± 1.34	1439 ± 3.54^a^

^a,b^Kruskal-Wallis test: values marked by letter symbols a, b,…, are statistically significant, *p* < 0.05 (values read in separate columns, marked with the same letter, do not differ significantly; those marked with different letters differ significantly).

**Table 3:** Polyphenol content, antioxidant activity DPPH, and color in the initial macerate in relation to the sage to fish oil ratio (*w*/*w*).

Sage to fish oil weight ratio	Polyphenols (mg/100 g)	DPPH activity (%)	Color
1/5.7	161.4 ± 5.464^a^	24.6 ± 1.539^a^	836 ± 3.536^a^
1/9	121.3 ± 1.518	16.7 ± 0.998	760 ± 1.414
1/12	103.5 ± 6.072^b^	14.1 ± 0.445^b^	599 ± 5.657^b^

^a,b^Kruskal-Wallis test: values marked by letter symbols a, b,…, are statistically significant, *p* < 0.05 (values read in separate columns, marked with the same letter, do not differ significantly; those marked with different letters differ significantly).

**Table 4:** Peroxide value, anisidine value, Totox of sage oil macerate after different maceration times, and cod liver oil (CLO_0_) used to obtain this macerate.

Maceration time (days)	Anisidine value (AV)	Peroxide value (PV) (meqO/1 kg)	Totox
0	1.10 ± 0.46	10.7 ± 0.09^b^	22.4 ± 0.65^b^
3	1.78 ± 0.55	11.0 ± 0.10^b^	23.9 ± 0.75^b^
6	2.80 ± 1.36	10.3 ± 0.02^b^	23.4 ± 1.31^b^
8	0.71 ± 0.40	16.1 ± 0.02^b^	32.9 ± 0.37^b^
10	0.97 ± 0.28	8.08 ± 0.05^b^	17.1 ± 0.09^b^
13	0.74 ± 0.77	12.4 ± 0.06^b^	25.6 ± 0.89^b^
15	2.53 ± 0.07	12.3 ± 0.07^b^	27.1 ± 0.07^b^
Average	1.52 ± 0.86	11.6 ± 2.47^b^	24.6 ± 4.82^b^
CLO	2.09 ± 0.43	6.03 ± 0.04^a^	14.1 ± 2.36^a^

^a,b^Kruskal-Wallis test: values marked by letter symbols a, b,…, are statistically significant, *p* < 0.05 (values read in separate columns, marked with the same letter, do not differ significantly; those marked with different letters differ significantly).

## Data Availability

The data used to support the findings of this study are available from the corresponding author upon request.
